# Vitamin D Ameliorates Impaired Wound Healing in Streptozotocin-Induced Diabetic Mice by Suppressing Endoplasmic Reticulum Stress

**DOI:** 10.1155/2018/1757925

**Published:** 2018-03-07

**Authors:** Yi Feng Yuan, Sushant K. Das, Mao Quan Li

**Affiliations:** Department of Intervention &Vascular Surgery, Shanghai Tenth People's Hospital, Tongji University School of Medicine, Shanghai, China

## Abstract

**Background:**

This study is designed to investigate whether vitamin D promotes diabetic wound healing and explore the potential mechanism which may be involved in the healing process.

**Material and Methods:**

Human umbilical vein endothelial cells (HUVECs) were treated with 200 *μ*g/ml of advanced glycation end product-modified human serum albumin (AGE-HSA) and 250 mg/dl of glucose with vitamin D. Cell viability was analyzed using the CCK-8 assay, and the apoptosis rate was measured using flow cytometry. Endogenous markers of ER stress were quantified using Western blot and a real-time polymerase chain reaction. Diabetic mice were treated with vitamin D (100 ng/kg per day) for 14 days. The ulcer area and ulcerative histology were detected dynamically.

**Results:**

Vitamin D administration not only decreased the apoptosis rate but also increased cell viability. Furthermore, the expression of endogenous markers of ER stress was downregulated as a result of vitamin D treatment. Vitamin D supplementation significantly accelerated wound healing of diabetic mice and improved the healing quality. Further studies showed that reduced ER stress was associated with the positive outcome.

**Conclusion:**

These results suggest that vitamin D may ameliorate impaired wound healing in diabetic mice by suppressing ER stress.

## 1. Introduction

Endothelial dysfunction plays a vital role in the pathogenesis and progression and complications of diabetic vascular diseases. In the past few decades, the effect of high glucose levels and advanced glycation end product on vascular endothelial cells has been widely studied. The mechanisms underlying endothelial dysfunction include accumulation of advanced glycation end products [1], increased oxidative stress [2], and endoplasmic reticulum (ER) stress [3]. Although antioxidants have been promoted and tested in clinical trials, antioxidants have not been shown to be effective in preventing vascular dysfunction. This finding may in part be due to the presence of ER stress, which is not influenced by antioxidant therapy [4].

The ER is a membrane-bound organelle responsible for several cellular functions, such as posttranslational folding of proteins and calcium storage. If ER homeostasis is disrupted, misfolded, or unfolded proteins within this organelle build-up (ER stress) [5]. The cellular response to ER stress is referred to as an unfolded protein response (UPR), which means translation is slowed down, protein degradation is induced, and the folding capacity of the ER is increased [6]. An UPR may act as a survival or apoptotic pathway depending on the severity of ER stress [7].

Vitamin D is known to enhance intestinal calcium absorption and increase the plasma calcium level [8]. According to recent studies, the biological effects of vitamin D extend far beyond calcium metabolism.

Mounting evidence and data suggest a link between vitamin D deficiency and an increased risk of cardiovascular diseases [9–15]. The low vitamin D status may be a contributing factor in peripheral arterial diseases and diabetic vascular diseases [16, 17]. Vitamin D induces biological effects by binding to its receptor (VDR) on target cells and organs. Activation of VDR impacts downstream genes to cause various effects, such as anti-inflammation, antiatherosclerosis, and direct cardioprotective actions [18–21].

This study is designed to investigate whether Vitamin D promotes diabetic wound healing and, furthermore, explore the potential mechanism which may be involved in the healing process.

## 2. Materials and Methods

### 2.1. Materials

The 1,25-dihydroxyvitamin D_3_ was purchased from Sigma-Aldrich (St. Louis, MO, USA). Fetal bovine serum, Dulbecco's modified Eagle's medium (DMEM)/F12, and trypsin were purchased from Hyclone (Logan, UT, USA). The CCK-8 assay was purchased from Dojindo (Japan). Annexin V-FITC/PI was purchased from Yeasen Corporation (Shanghai, China). A PrimeScript RT Reagent Kit and SYBR Premix Ex Taq™ II were purchased from TaKaRa (Tokyo, Japan). TRIzol reagent was purchased from Invitrogen (Carlsbad, CA, USA). Primers targeting glucose-regulated protein (GRP) 78, ATF4, CHOP, VDR, and GAPDH were synthesized by Sangon Biotech (Shanghai, China). Radio immunoprecipitation assay (RIPA) lysis buffer, a bicinchoninic acid (BCA) protein assay kit, antibody solution, and 5x sodium dodecyl sulfate-polyacrylamide gel electrophoresis (SDS-PAGE) same loading buffer were purchased from Beyotime Biotechnology (Shanghai, China). Antibodies (VDR, PERK P-PERK, eIF-2*α*, P-eIF-2*α*, ATF4, CHOP, and GRP78) were purchased from Cell Signaling Technology (Beverly, MA, USA). Polyvinylidene difluoride membranes (0.45 mm) were purchased from Millipore (Billerica, MA, USA). Human serum albumin (HSA; 1.5 mmol/l) was purchased from Sigma-Aldrich.

### 2.2. Experimental Design

Human umbilical vein endothelial cells (HUVECs) were divided into four groups based on treatment, as follows: 200 *μ*g/ml of HSA and 100 mg/dl of glucose (normal group); 200 *μ*g/ml of AGE-HSA and 250 mg/dl of glucose (diabetic group); 200 *μ*g/ml of AGE-HSA and 250 mg/dl of glucose with vitamin D (50 ng/ml) (VD treatment group); and 200 *μ*g/ml of AGE-HSA, 250 mg/dl of glucose, vitamin D (50 ng/ml), and VDR siRNA (VD antagonist group). Each experiment was repeated at least three independent experiments.

### 2.3. Cell Culture

HUVECs were obtained from Shanghai Bogoo Biotechnology (Shanghai, China). Cells were incubated in DMEM/F12 containing 5 mM d-glucose at 37.0°C in a humidified atmosphere of 5% CO_2_ in air until 80% confluence. The cells were exposed to different conditions. Medium was replaced every 2-3 days and 24 h before the end of the experiment.

#### 2.3.1. Inhibition of VDR Expression via siRNA Silencing

To knock out VDR expression, HUVECs were transfected with 10 nM control siRNA or 10 nM VDR-specific siRNA using lipofectamine (Invitrogen, Carlsbad, CA, USA) for 72 h. VDR levels were then measured by Western blotting.

### 2.4. Cell Viability

Cell viability was measured using the CCK-8 assay, following the manufacturer's protocol. Briefly, HUVECs were placed in 96-well plates at 5000 cells/well. The plates were preincubated at 37.0°C in a humidified atmosphere of 5% CO_2_ in air until 80% confluence. Then, 10 *μ*l of the CCK-8 solution was added to each well of the plate. The plate was incubated for 1–4 h. The absorbance was measured at 450 nm using a microplate reader.

### 2.5. Cell Apoptosis

Cell apoptosis was detected by flow cytometry. Cells were collected after treatment and washed twice with cold phosphate-buffered saline. Then, the cells were resuspended at 1 × 10^6^/ml in 500 *μ*l of binding buffer, which was included in the Annexin V-FITC/propidium iodide(PI) apoptosis detection kit and treated with 5 *μ*l of Annexin V-FITC and 5 *μ*l of PI following the manufacturer's protocol. Subsequently, the cells were incubated for 10 min before the apoptosis rate was measured by flow cytometry.

### 2.6. RNA Extraction and RT-PCR

Total RNA was extracted from HUVECs with TRIzol reagent following the manufacturer's protocol, and a PrimeScript RT Reagent Kit was used to synthesize complementary DNA. RT-PCR was performed to validate the expression pattern of selected genes (*VDR*, *GRP78*, *CHOP*, and *ATF4*) using SYBR Premix EX Taq II. Data were analyzed using the 2^−∆∆Ct^ method. *GAPDH* was used as a reference gene. The primers used are listed in Table 1.

### 2.7. Western Blot Analysis

HUVECs were washed three times with cold phosphate-buffered saline after treatment. Cells were collected and lysed for 30 min on ice in RIPA buffer containing a protease inhibitor cocktail before being centrifuged at 12,000 rpm for 15 min at 4°C to obtain the protein-containing supernatant. The protein concentration was then measured with a BCA assay kit. The proteins were separated by 10%–12% SDS-PAGE and transferred to a 0.45 *μ*m polyvinylidene difluoride membrane, which was then blocked in 5% skim milk for 1 h at room temperature, and incubated with primary antibodies at 4°C overnight. The membranes were then washed three times with Tris-buffered saline Tween 20 and incubated with horseradish peroxidase-conjugated secondary antibodies for 1 h at room temperature. The expression of various proteins was subsequently visualized by enhanced chemiluminescence.

#### 2.7.1. Animals and Induction of DM

6-week old male ICR mice were purchased from SLAC Laboratory Animal Co. Ltd. (Shanghai, China) and used to establish a mouse model for DM. All mice were lodged in individual cages in a temperature- and humidity-controlled room (22 ± 1°C and 50 ± 1% humidity) with 12-hour light cycle in the animal facility of the Animal Unit of Tongji University. Diabetes was inducted in mice with STZ injected intraperitoneally once at a dose of 100 mg/kg (in 0.01 M sodium citrate, pH 4.3–4.5). Nondiabetic mice were injected with only a saline vehicle. After 7 days, mice with fasting blood glucose levels higher than 250 mg/dl were considered as diabetic. All animal experiments were conducted in accordance with the National Institutes of Health Guide for the Care and Use of Laboratory Animals and approved by the Biological Research Ethics Committee of the Chinese Academy of Sciences. Forty-five mice were randomly divided into three groups, as follows: (1) normal group: nondiabetic mice who received a saline vehicle for 14 days; (2) diabetic mellitus group (DM group): diabetic mice were injected with a saline vehicle for 14 days; and (3) vitamin D treatment group (VD treatment group): diabetic mice were treated with vitamin D (100 ng/kg per day) for 14 days.

#### 2.7.2. Wound Biopsy and Measurement of Wound Closure

After 4 weeks of STZ induction, a model for diabetic wound was created in mice as follows: All mice were anesthetized with isoflurane, and their back were shaved and sterilized. Then full thickness excisional wounds were made on the dorsal back by a disposable 6 mm skin biopsy punch and Westcott scissor. Wounds in individual mice were photographed digitally days 0, 3, 6, and 9 until the end (day 14). A digital camera (EOS50; Cannon, Japan) was employed to take pictures, and the ulcer area was analyzed by Image-Pro Plus 4.5 software.

#### 2.7.3. Histological Assessment of Wound Healing

The wounds, together with unwounded skin margins, were collected in both groups, fixed in paraffin, and sectioned at 5.0 *μ*m. The sections were dehydrated with successive concentrations of ethanol and washed twice in distilled water. The sections of the ulcerative tissue at D14 were stained with hematoxylin and eosin (H&E) and with Masson's trichrome in accordance with the protocols of the manufacturer (Cyagen Biosciences Inc.) to detect the reepithelialization/granulation tissue formation and collagen deposition, respectively.

#### 2.7.4. Measurement of ER Stress in Diabetic Wounds

Mouse wounds were harvested and homogenized in cold PBS supplemented with protease inhibitor cocktail (Sigma-Aldrich) by using a Dounce homogenizer and then sonicated and centrifuged at 10000 rpm for 20 minutes at 4°C. Supernatants were used for RNA extraction. RT-PCR was performed to validate the expression pattern of selected genes (*GRP78*, *CHOP*, and *ATF4*) using SYBR Premix EX Taq II. Data were analyzed using the 2^−∆∆Ct^ method. *GAPDH* was used as a reference gene.

### 2.8. Statistical Analysis

All cell data are expressed as the mean ± SD of at least three independent experiments. Each in vivo experiment was conducted using at least fifteen mice per group. Results are also expressed as mean ± SD. Differences between the experimental groups were assessed by Student *t*-test or one-way analysis of variance, followed by Dunnett's test. For all statistical analyses, *p* values < 0.05 were considered statistically significant.

## 3. Results

### 3.1. Cell Experiment

#### 3.1.1. Knockout VDR Expression in HUVECs

**Figure 1:**
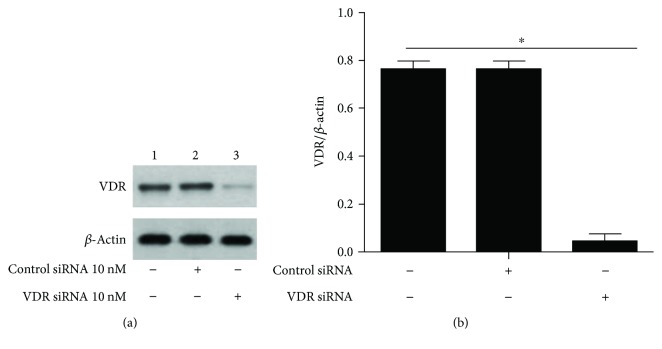
siRNA-mediated VDR knockout in HUVEC. (a) HUVECs were transfected with either control siRNA (10 nM) or VDR siRNA (10 nM), and VDR expression was measured 72 h later by Western blot. (b) Western blot was quantified, and VDR expression is presented as percentage. ^∗^*p* < 0.05, relative to normal cells and cells transfected with control siRNA.

HUVECs were transfected with 10 nM control siRNA or VDR siRNA for 72 h, and then VDR expression was measured by Western blotting (Figure 1). VDR expression decreased by 72.4% compared with the blank and control groups. Based on these results, we confirmed that 10 nM VDR siRNA successfully knocked down VDR expression.

#### 3.1.2. Effect of Vitamin D on Cell Viability and Apoptosis

**Figure 2:**
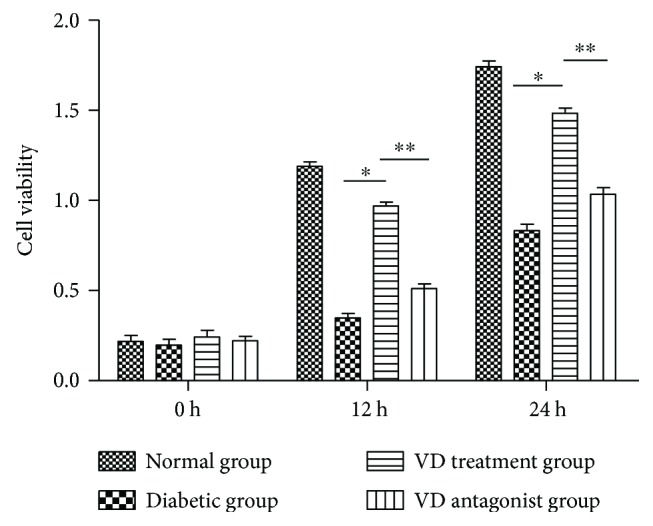
Effect of vitamin D on cell viability: HUVECs were exposed to different conditions for 0, 12, and 24 h. Normal group (200 *μ*g/ml human serum albumin (HAS) and 100 mg/dl glucose); diabetic group (200 *μ*g/ml AGE-HSA and 250 mg/dl glucose); vitamin D treatment group (vitamin D 50 nm, 200 *μ*g/ml AGE-HSA, and 250 mg/dl glucose); and vitamin D antagonist group (vitamin D 50 nm, VDR siRNA 10 nm, 200 *μ*g/ml AGE-HSA, and 250 mg/dl glucose). Cell viability was measured by CCK-8 assay. ^∗^*p* < 0.05 relative to the VD treatment group. ^∗∗^*p* < 0.05 relative to the VD antagonist group.

**Figure 3:**
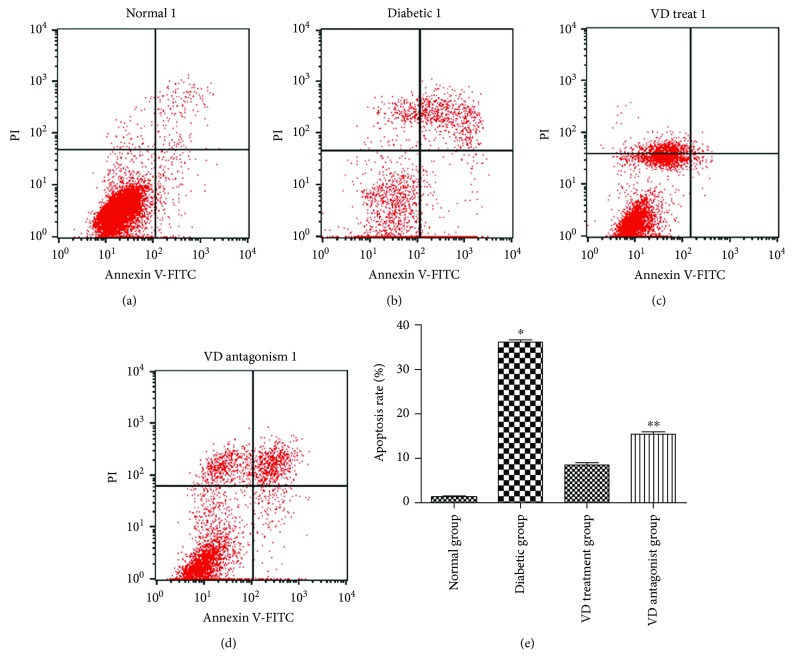
Effect of vitamin D on cell apoptosis. HUVECs were exposed to different conditions. Cell apoptosis rate was measured by flow cytometry. (a) Normal group. (b) Diabetic group. (c) Vitamin D treatment group. (d) Vitamin D antagonist group. (e) Apoptosis rate in each group. ^∗^*p* < 0.05 relative to the VD treatment group. ^∗∗^*p* < 0.05 relative to the VD treatment group.

HUVECs were incubated for various lengths of time (0 h, 12 h, and 24 h) under different conditions. The cell viability in each group was measured by the CCK-8 assay. As shown in Figure 2, the cell viability in the diabetic group was sharply diminished compared with the normal group. This reduction was reversed by pretreatment with vitamin D. The cell viability in the vitamin D group was similar to the normal group; thus, vitamin D has the potential to restore endothelial cell dysfunction. Interestingly, coadministration of vitamin D and VDR siRNA abolished the restoration ability; thus, the VDR pathway was involved in the process. A similar outcome was observed by detection of cell apoptosis in different groups (Figure 3). The cell apoptosis rate was measured with an Annexin V-FITC/PI assay using flow cytometry. The apoptosis rate in the diabetic group was significantly higher than the normal group. In the vitamin D group, the endothelial cell apoptosis rate decreased. This positive effect was blocked by the VDR suppressor.

#### 3.1.3. The Expression of Endogenous Biomarkers of ER Stress

**Figure 4:**
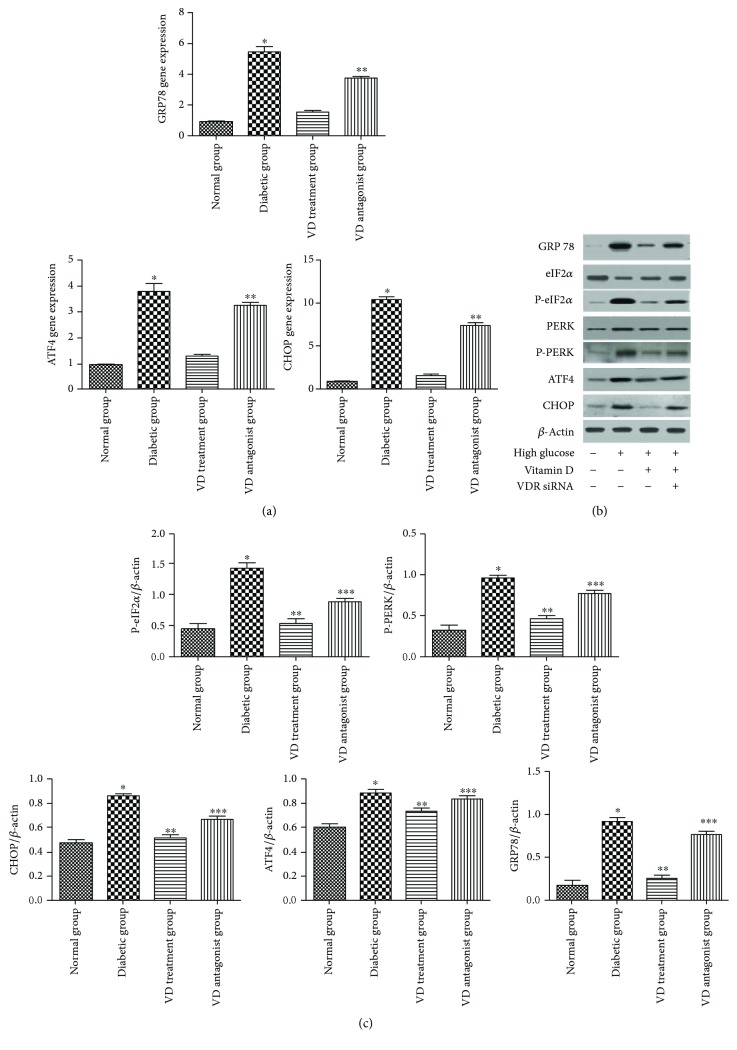
Effect of vitamin D on endoplasmic reticulum stress. (a) mRNA levels of GRP78, ATF4, and CHOP. ^∗^*p* < 0.05, relative to the VD treatment group. ^∗∗^*p* < 0.05 relative to the VD treatment group. (b, c) Protein levels of GRP78, PERK, P-PERK, eIF-*α*, P-eIF-*α*, ATF4, and CHOP. Expression is presented as percentage. ^∗^*p* < 0.05, relative to the normal group. ^∗∗^*p* < 0.05, relative to the diabetic group. ^∗∗∗^*p* < 0.05, relative to the VD treatment group.

ER stress is a new mechanism underlying the pathogenesis of endothelial dysfunction induced by a diabetic-like environment. In the current study, the endogenous biomarkers of ER stress were detected at the gene and protein levels (Figure 4). The diabetic-like environment induced GRP78 expression relative to the normal group, which was inhibited by treatment with vitamin D for 24 h. Activation of GRP78 enabled dimerization of PERK and its consequent auto-phosphorylation and then phosphorylated downstream eIF2a. Both phosphorylated PERK and eIF2a were inhibited by vitamin D treatment. The diabetic-like environment had no effect on nonphosphorylated PERK and eIF2a. As PERK-eIF2a-ATF4-CHOP represents a classical ER stress pathway, the expression of ATF4 and CHOP was detected by PCR and Western blotting. It was shown that a diabetic-like environment also induced ATF4 and CHOP expression. In the vitamin D group, ATF4 and CHOP expression was decreased. Conversely, the increased expression of ATF4 and CHOP was detected in the VDR antagonist group.

#### 3.1.4. VDR Expression in Different Groups

**Figure 5:**
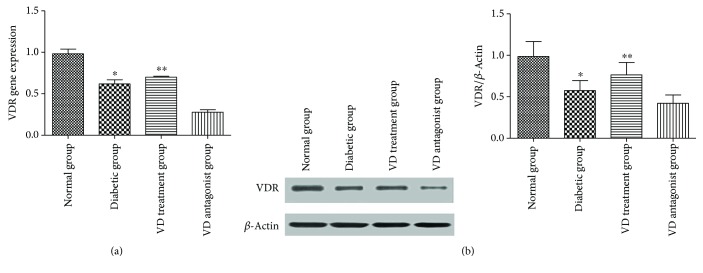
VDR expression in different groups: (a) mRNA levels of VDR gene in different groups. (b) Western blot was quantified, and VDR expression is presented as percentage. ^∗^*p* < 0.05, relative to the normal group. ^∗∗^*p* > 0.05, relative to the diabetic group.

VDR expression was detected in different groups. RT-PCR was used to analyze the difference in VDR gene expression in each group. The results showed that VDR expression was decreased in the diabetic group compared with the normal group. Vitamin D treatment partly restored VDR expression (Figure 5(a)). There was no statistical significance between the diabetic and VD treatment groups. A similar outcome was confirmed by Western blot analysis (Figure 5(b)).

#### 3.1.5. Animal Experiment

**Figure 6:**
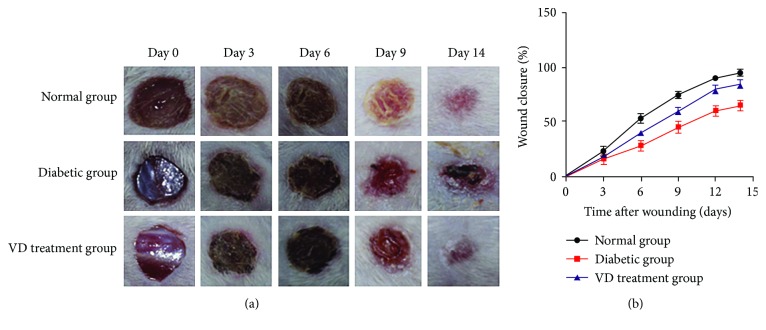
Vitamin D treatment accelerated diabetic wound healing. (a) 6 mm diameter wounds were created by punch biopsy, and the closure of the wound area was measured by digital camera every 3 days until day 14. (b) Percentage of wound closure (means ± SD). Healing of diabetic wounds significantly delayed compared with normal wounds. Vitamin D began to improve diabetic wound closure on day 6. At the end of the observation (day 14), the VD treatment group exhibited improved wound healing, compared with the diabetic group.

*(1) Supplementation of Vitamin D Accelerated Diabetic Wound Healing*. The representative ulceration images for the three groups at D0, D3, D6, D9, and D14 posttreatment are presented in Figure 6.  Figure 6(b) shows the mean area of ulceration at relevant time points. At D3 posttreatment, incrustation was formed in each group; there was no significant difference to the size of ulcers. By the end of observation (D14), nondiabetic wounds completely healed, while most of the diabetic wounds remained open with a low average closure rate of 65%. Vitamin D significantly improved diabetic wound closure and increased the healing rate of diabetic wound by 20.4% (85.4% versus 65.0%, *p* < 0.05).

**Figure 7:**
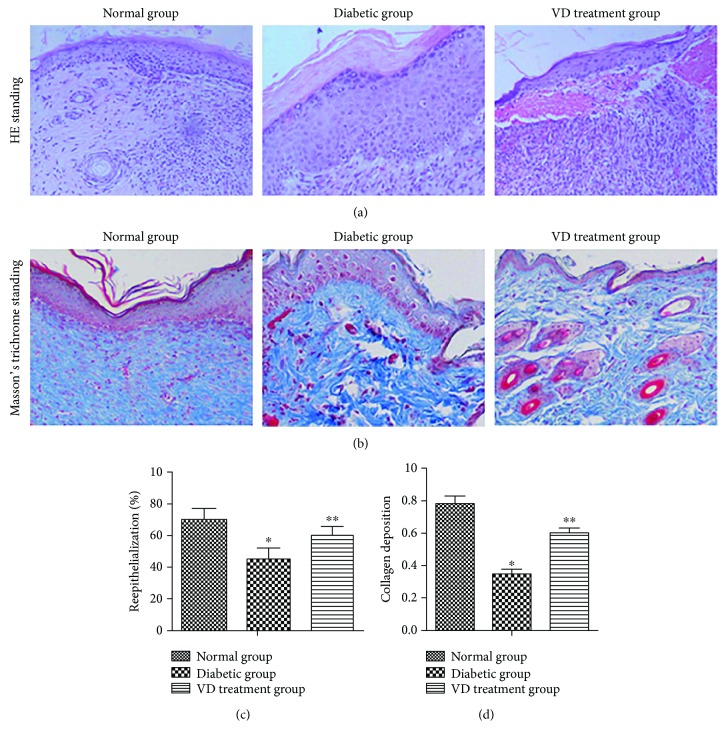
Effects of vitamin D on the epithelialization and collagen deposition in ulcerative tissues at D14 after treatment. (a) H&E staining of sections showed better dermal reepithelialization on the diabetic wounds in the VD treatment group compared with the diabetic group. (b) Collagen deposition assessed by Masson's trichrome staining. (c, d) Statistical reepithelialization and thickness of collagen deposition of wounds by computer-assisted morphometric analysis. Data are represented as means ± SD. ^∗^*p* < 0.05, compared with the normal group. ^∗∗^*p* < 0.05, compared with the diabetic group.

*(2) Supplementation of Vitamin D Improved Diabetic Wound Healing*. Reepithelialization was measured at day 14 after wounding by the histomorphometric analysis of sections stained with HE. As shown in Figure 7(a), at day 14 after wounding, the wound was not fully reepithelialized in the DM group, while the wound got close to fully reepithelialized in the normal group. In the VD treatment group, the epithelia were significantly longer compared with the DM group.  Figure 7(b) shows that collagen formation in the ulcer tissues at D14 was assessed by Masson's trichrome staining using computer-assisted morphometric analysis. Less amount of collagen deposition organized in aligned fibers in the diabetic groups, but relatively more in the normal group (0.35 ± 0.026 versus 0.78 ± 0.045, *p* < 0.05). Interestingly, vitamin D supplementation improved collagen deposition in ulcer tissues compared with the DM group. The mean value of the VD treatment group was 0.60 ± 0.031, and for the DM group was 0.35 ± 0.026 (*p* < 0.05).

**Figure 8:**
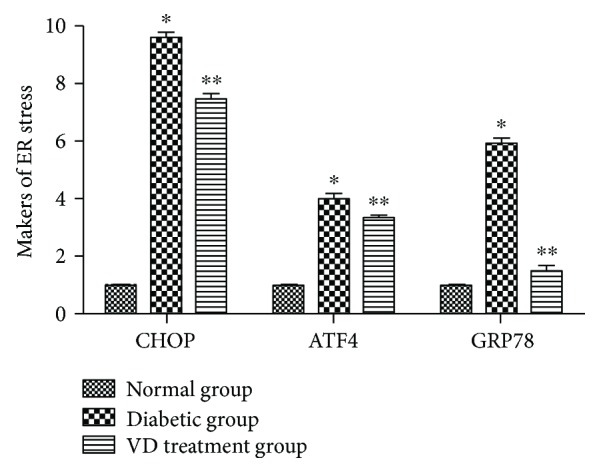
mRNA levels of GRP78, ATF4, and CHOP in diabetic wound tissues. ^∗^*p* < 0.05, relative to the normal group. ^∗∗^*p* < 0.05 relative to the VD treatment group.

*(3) The Expression of Endogenous Biomarkers of ER Stress in Diabetic Wounds*. As shown in Figure 8, the markers of ER stress (*GRP78*, *CHOP*, and *ATF4*) were significantly increased in the diabetic group, compared with the normal group. With Vitamin D supplementation, ER stress markers were markedly reduced in diabetic wound tissues, compared with those in the diabetic group.

## 4. Discussion

Vitamin D is well known as a regulator of epidermal and hair follicle differentiation. Tian et al. observed that topical 1,25(OH)_2_D enhanced wound healing [22]. Luderer et al. observed that in the global vitamin D receptor (VDR) knockout mouse, there was a reduction in TGF-*β* signaling in the dermis [23]. Oda et al. observed that reepithelialization is impaired when the deletion of VDR is accompanied by a low calcium diet [24]. Besides that, several studies documented an anti-inflammatory effect of vitamin D in variety of cell types, including endothelial cells [25, 26], dentritic cells [27, 28], T cells [29], and macrophages [30], which was in part linked to an inhibition of NF-*κ*B activation and signaling [31]. Mounting researches observed that vitamin D supplementation has positive effect on diabetic wound healing. But the true mechanism is still uncertain, especially the relationship between vitamin D and ER stress.

ER stress promotes endothelial dysfunction and diabetic vascular disease. When ER homeostasis is disrupted, the UPR pathway is activated. The UPR, known to be a crucial defensive mechanism in ER stress, is mediated by three transmembrane proteins (IRE1, protein kinase R-like endoplasmic reticulum kinase, and activating transcription factor 6). Under normal conditions, these proteins are maintained in an inactive state when bound to GRP78, a chaperone considered to be an indicator of ER stress. When homeostasis is disrupted, GRP78 dissociates from membrane proteins, leading to activation of three corresponding pathways [32].

The PERK axis is one of the ER stress pathways. Release of GRP78 upon build-up of misfolded proteins, inadequate glucose levels, or other stressors enable dimerization of PERK and its consequent auto-phosphorylation. Activated PERK then phosphorylates the alpha subunit of eIF2, leading to global translation arrest. Transcripts with alternative upstream open reading frames, such as ATF4, are then preferentially translated. Expression of ATF4 can lead to induction of CHOP. Upon severe and sustained ER stress, CHOP promotes apoptosis.

In the present study, vitamin D supplementation not only partly restores dysfunction of endothelial cells which were exposed to diabetic-like environments but also improves impaired wound healing in streptozotocin-induced diabetic mice. ER stress pathway is involved in both phenomenons. It is reasonable to presume that Vitamin D ameliorates impaired wound healing in streptozotocin-induced diabetic mice by suppressing endoplasmic reticulum stress.

Whether vitamin D could improve other abnormalities leading to diabetic ulcer, such as vascular diseases and neuropathies, will be a next job in our further research work. And whether this drug can be extrapolated to clinical situations still needs further investigation in clinical trials.

## Figures and Tables

**Table 1:** The primes of ER stress genes.

GRP78	Forward: 5′-TAGCGTATGGTGCTGCTGTC-3′
	Reverse: 5′-CCTTGGAATCAGTTTGGTCAT-3′

CHOP	Forward: 5′-GCCTTTCTCCTTTGGGACACTGTCCAGC-3′
	Reverse: 5′-CTCGGCGAGTCGCCTCTACTTCCC-3′

ATF4	Forward: 5′-GGAGGTGGCCAAGCACTTCA-3′
	Reverse: 5′-CTTCTGGCGGTACCTAGTGG-3

VDR	Forward: 5′-AGGCGAAGCATGAAGCGGAAG-3′
	Reverse: 5′-GCGTCCAGCAGTATGGCAATGA-3′

GAPDH	Forward: 5′-CCAGCAAGAGCACAAGAGGAA-3′
	Reverse: 5′-ATGGTACATGACAAGGTGCGG-3′
